# Annotations

**Published:** 1909-06-26

**Authors:** 


### ANNOTATIONS.

### The Medical Defence Societies.

Once more the three societies which exist—two in England and one in Scotland—for the purpose of protecting members of the profession from the frequently fraudulent and almost always unfounded claims at law of litigious patients or covert blackmailers, have a year of steady and unchecked progress to report. One of the most gratifying features about all three reports is the uniform increase in membership. The Medical and Dental Defence Union of Scotland obtained 240 new members during the year; the London and Counties Medical Protection Society 340, and the Medical Defence Union 478: compared with these additions to the rolls, the losses by death and resignation were small, and each Society made a substantial gain. With the exception of the first-named possibly, the increases were smaller than has frequently been the case in the past; but the fact of the matter doubtless is that so large a proportion of the profession now belongs to one or other Society, that the field for recruiting is becoming much more limited. Law costs, it is apparent from the annual reports, tend to become heavier each year, and absorb the lion's share of the subscriptions to the Societies, which are, however, all living within their incomes. To build up a reserve fund of investments against a possible run of misfortunes and adverse verdicts is obviously a wise and prudent course: yet at the same time there is no object in unduly increasing the resources of defence corporations, which are not intended to be profit-making concerns, but are really systems of mutual insurance between the members. From this point of view, therefore, the fact that income is nearly balanced by expenditure is a sign of a sound financial position; and the smallness of the surplus is not really any cause for disquietude. It is also noticeable how seldom it is possible to recover costs when awarded in courts of law against the litigants whose obstinacy or perversity forces the Societies to take up cases on behalf of members. The man of straw seems to be particularly prominent in attacks on the medical profession—another proof, if any were needed, of the unwisdom of the practitioner who remains outside the membership of one or other of these beneficent institutions.

### The Menace of Cholera.

A laconic telegram in the foreign news column of the daily papers announces that on Saturday last there were notified fifty-three cases of cholera in St. Petersburg, and ten deaths. Without wishing in any way to sound a note of panic or needless alarm, we do seriously direct the attention of the medical profession to this announcement, the full import of which lies perhaps a little below the surface. It will be remembered that the disastrous epidemic of last autumn was brought to a close, in accordance with the forecasts of experts, by the advent of winter and the freezing in of the Neva and the Petersburg canals. It was further predicted that, unless the public health authorities and sanitary scientists of the Tsar's capital could devise efficient prophylaxis in the meantime, a very serious risk of a recrudescence of the scourge might be looked for when the ice should melt. The Baltic opens as a rule in May, and already cholera cases have risen to the figures quoted above. These figures are, of course, small compared with those of last October, but it has to be remembered that the epidemic of 1908 did not begin until the beginning of September: it seems only too likely that, with the hot, dry months still ahead, St. Petersburg may be as heavily visited as last year, or even more disastrously still. What concerns this country especially is the risk of the importation of the disease by the crews of the very numerous vessels plying in the timber and other trades between the two countries. We cannot afford to look with equanimity upon any outbreak of this kind in a city whose commercial relations with Hull and other English seaports are so intimate. Still, forewarned is forearmed; and it cannot be said, in the event of any cholera cases making their appearance in this country, that there has not been ample indication beforehand of the quarters in which danger lies. It is to be hoped that no such cases will occur, but the contingency is not impossible.

### Embankment Pests in Relation to the Public Health.

The miserable plight of those outcasts of humanity who frequent the Thames Embankment by night, and endeavour to sleep upon the seats there, is a theme which has for years supplied novelists, journalists, and dramatic authors with material for effective contrast with the luxury and glamour of the big hotels overlooking the river. There is, however, an aspect of the situation which is untouched as a rule by those in quest of an antithesis or a moral; and that is the relation of these persons who spend their nights on the Embankment to the public health. There is little doubt that—brilliant exceptions notwithstanding—the ordinary Embankmenter is a person whose character is irredeemable. Drink and disease and other kindred forces of evil have submerged his moral nature to a depth from which not even the ministrations of the most practical philanthropy can lift him: vermin swarm upon him, and of dirt-disease he is a prolific source. Now the seats on the Embankment are used during the day by ordinary citizens of various degree, and especially by those to whom the State is beginning to recognise its obligations, the children. And there can be no possible defence of a system which allows verminous and frequently infective persons to spend the night on public seats which healthy people innocently use during the day. It appears from the evidence given at an inquest held the other day upon one of these miserable pests that the Cleansing of the Person Act, which is optional for local authorities, confers no power on the police to arrest and compel the disinfection of the repulsive hives of vermin, who, according to the police, swarm nightly upon what might be one of London's most admired promenades. One thing or the other—either compulsion for local authorities or extension of the powers of the police—seems urgently called for by a scandal to which it is incredible that a city like London should remain indifferent.

